# The *Bacteroidetes* Q-Rule: Pyroglutamate in Signal Peptidase I Substrates

**DOI:** 10.3389/fmicb.2018.00230

**Published:** 2018-03-01

**Authors:** Matthias Bochtler, Danuta Mizgalska, Florian Veillard, Magdalena L. Nowak, John Houston, Paul Veith, Eric C. Reynolds, Jan Potempa

**Affiliations:** ^1^International Institute of Molecular and Cell Biology, Warsaw, Poland; ^2^Institute of Biochemistry and Biophysics, Polish Academy of Sciences, Warsaw, Poland; ^3^Department of Microbiology, Faculty of Biochemistry, Biophysics, and Biotechnology, Jagiellonian University, Krakow, Poland; ^4^Department of Oral Immunology and Infectious Diseases, University of Louisville School of Dentistry, Louisville, KY, United States; ^5^Oral Health Cooperative Research Centre, Melbourne Dental School, Bio21 Institute, The University of Melbourne, Melbourne, VIC, Australia

**Keywords:** *Bacteroidetes*, signal peptidase I, glutaminyl cyclase, pyroglutamate, *Porphyromonas*

## Abstract

*Bacteroidetes* feature prominently in the human microbiome, as major colonizers of the gut and clinically relevant pathogens elsewhere. Here, we reveal a new *Bacteroidetes* specific feature in the otherwise widely conserved Sec/SPI (Sec translocase/signal peptidase I) pathway. In *Bacteroidetes*, but not the entire FCB group or related phyla, signal peptide cleavage exposes N-terminal glutamine residues in most SPI substrates. Reanalysis of published mass spectrometry data for five *Bacteroidetes* species shows that the newly exposed glutamines are cyclized to pyroglutamate (also termed 5-oxoproline) residues. Using the dental pathogen *Porphyromonas gingivalis* as a model, we identify the PG2157 (also called PG_RS09565, Q7MT37) as the glutaminyl cyclase in this species, and map the catalytic activity to the periplasmic face of the inner membrane. Genetic manipulations that alter the glutamine residue immediately after the signal peptide in the pre-pro-forms of the gingipains affect the extracellular proteolytic activity of RgpA, but not RgpB and Kgp. Glutamine statistics, mass spectrometry data and the mutagenesis results show that the N-terminal glutamine residues or their pyroglutamate cyclization products do not act as generic sorting signals.

## Introduction

Bacteria of the *Bacteroidetes* phylum of gram-negatives live in very diverse habitats including human hosts (Thomas et al., 2011). Studies of the diversity of the healthy human microbiome in various tissues have shown that the *Bacteroidetes* colonize many human tissues. In the gut, they are particularly abundant, and together with the *Firmicutes*, they comprise over 90% of the microbiome in Western populations (Human Microbiome Project Consortium, 2012; Johnson et al., 2017). Outside the gut, many *Bacteroidetes* species may cause significant clinical problems (Wexler, 2007). This is best illustrated by chronic periodontitis, the infection-driven inflammation of tooth-supporting tissues initiated and propagated by *Porphyromonas gingivalis* and *Tannerella* *forsythia* colonizing subgingival tooth surface (Holt and Ebersole, 2005; How et al., 2016). Protein secretion has been found to be essential for these species for interactions with the host, in part, but not only, because many identified virulence factors are secreted proteins (Green and Mecsas, 2016).

Apart from the Sec pathway that imports proteins into or through the inner membrane (Pugsley, 1993; Facey and Kuhn, 2010), the repertoire of well-characterized secretion pathways of *Bacteroidetes* is surprisingly limited. *Bacteroidetes* possess type 1 and type 6 secretion systems (T1SSs and T6SSs) that export proteins bypassing the periplasm. In recent years, much effort has been invested to characterize the T9SS, which is typical for *Bacteroidetes* and rarely if ever found outside the phylum (Abby et al., 2016; Lasica et al., 2017). The T9SS transports proteins that reach the periplasm via the Sec translocon and possess a characteristic C-terminal domain (CTD) through the outer membrane (Veith et al., 2013; Lasica et al., 2017). The steps upstream of CTD-dependent secretion have attracted less attention, because they are generally not unique to *Bacteroidetes* and have been extensively studied in gram-negative bacteria in general (Pugsley, 1993). In this work, we report that the upstream steps have unique features in *Bacteroidetes* that merit attention. In order to put our work into perspective, some background on the Sec pathway is helpful.

The Sec pathway identifies client proteins by the presence of a signal peptide (Auclair et al., 2012). Signal peptides have a tripartite architecture. They consist of an N-terminal positively charged region, thought to target proteins to the phospholipid membrane, a hydrophobic region, thought to be inserted into the membrane, and a short region that frequently contains a consensus motif for a signal peptidase (von Heijne, 1985; Paetzel et al., 2002). Proteins that carry signal peptides can escape in two different ways from the Sec translocase. If they escape “laterally,” they become embedded in the inner membrane (Facey and Kuhn, 2010). Otherwise, they reach the periplasm, but the signal peptide remains in the inner membrane, with its C-terminal end (with a signal peptide cleavage site) exposed on the periplasmic surface of the inner membrane (Paetzel et al., 2002). The further fate of proteins that have reached this stage depends on the type of the signal peptide.

Proteins that carry a type I signal peptide are released from their membrane anchored signal peptide by signal peptidase I (SPI) (Paetzel et al., 2002; Auclair et al., 2012). They can then remain in the periplasm, or be transported further, for example by a T9SS through the outer membrane, for an ultimate destination on the surface of the outer membrane, or for release into the medium (Lasica et al., 2017). Proteins that carry a type II signal peptide are processed differently. In a first step, a diacylglyceryl transferase (termed Lgt) attaches a diacylglycerol membrane anchor (in thioether linkage) to the cysteine residue immediately downstream of the signal peptide (Sankaran and Wu, 1994). The lipoprotein can then be cleaved just upstream of the modified cysteine residue by the signal peptidase II (SPII) (also known as lipoprotein signal peptidase or Lsp) (Yamagata et al., 1983; Hayashi and Wu, 1990). Some lipoproteins remain attached to the inner membrane while others are transported to the outer membrane via the Lol system (Okuda and Tokuda, 2011). Thus, lipoproteins in gram-negative bacteria are generally periplasmic proteins anchored to the inner or outer membrane.

The N-terminal residue of proteins is frequently chemically modified, and often such modifications have a signaling role. If an N-terminal glutamine residue is exposed as a result of proteolysis, this glutamine residue has a tendency to cyclize to pyroglutamate, with release of ammonia as a side product. The transamidation reaction is thought to initiate with nucleophilic attack of the α-amino group on the carbonyl group of the side chain carboxamide, followed by collapse of the oxyanion intermediate and protonation of the leaving ε-amino group. Glutamine cyclization to pyroglutamate can occur spontaneously. The reaction is facilitated by inorganic catalysts such as phosphate ions serving as the proton shuttle, or can be catalyzed enzymatically by glutaminyl cyclases (QCs) (Seifert et al., 2015).

Glutaminyl cyclases (QCs) have been identified in prokaryotes and eukaryotes. Broadly, they fall into two evolutionarily unrelated classes, represented by the well-studied human enzyme (HsQC) on the one hand and the enzyme from the plant *Carica papaya* (CpQC) or the related enzyme from the plant pathogen *Xanthomonas campestris* (XcQC) on the other hand. HsQC shares the α/β-hydrolase fold and active site architecture with bacterial Zn^2+^ dependent amino peptidases. D159, E202 and H330 bind the catalytic Zn^2+^ ion. E201 (in its anionic form) and D248 (in its neutral form) are thought to activate the nucleophilic α-amino group, and to promote departure of the ε-amino group, respectively. The superfamily of HsQC-like enzymes contains both QCs and aminopeptidases, with the same set of conserved active site residues (E201 and D248 catalyze proton transfer in the QCs and bind an additional metal cation in the aminopeptidases). From conservation of active site residues alone, QC activity can therefore not necessarily be inferred for the superfamily. The plant CpQC and the related bacterial XcQC adopt a five-bladed beta-propeller fold (Wintjens et al., 2006). As expected for beta-propeller proteins, the active site is located near the propeller axis (Russell et al., 1998). The mechanism of catalysis is much less clear than for HsQC. The well-conserved E69 is thought to serve as a proton shuttle, and K225 and N155 may be involved in oxyanion stabilization. In the case of plant QC enzymes, we are not aware of any reports of alternative aminopeptidase activity. Conservation of active site residues is therefore strongly suggestive of QC activity.

The presence of proteins containing N-terminal pyroglutamate residues has been noted in previous proteomic studies on *Bacteroidetes* species (Veith et al., 2002, 2009, 2013, 2014, 2015). At the outset of this study, we became aware of a drastic overrepresentation of glutamine residues after SPI cleavage sites in *P. gingivalis*. Starting from these two observations, we aimed to (1) clarify the pathway of pyroglutamate formation, (2) determine the fraction of SPI substrates that could be cyclized, (3) estimate the fraction that is actually cyclized, and (4) test a possible role of pyroglutamate formation in sorting, initially for *P. gingivalis* only, and then for *Bacteroidetes* in general.

## Materials and Methods

Proteomes and taxonomy information were taken from UNIPROT (The UniProt Consortium, 2017). Signal peptides and cleavage sites were predicted using the batch version of SIGNALP4.1 (Petersen et al., 2011). Lipoproteins were predicted using LipoP1.0 (Juncker et al., 2003). Sequence logos were generated using Weblogo (Crooks et al., 2004). Intersections between proteins with a predicted signal peptide and predicted lipoproteins were using the UNIX comm tool. Unfortunately, not all UNIPROT species are fully identified, and in some cases, species names such as “Tannerella sp.” were encountered. Proteins with such designations were pooled into “superspecies”, with particularly well-defined *Q*-values, and an anomalously large number of predicted proteins with predicted signal peptides or type I signal peptides. In the analyzed species groups, the “superspecies” constituted only 15% or less, except in the Cyanobacteria, where 31% of species were incompletely designated, often without clear signs that several species were pooled (judging from the number of proteins). Including the “superspecies”, 337 *Bacteroidetes* species, 13 *Chlorobi* species, 59 *Spirochaetes* species, 13 *Chlamydia* species, and 82 *Cyanobacteria* species were analyzed. The number of proteins with predicted signal peptides was above 50 in all analyzed species, guaranteeing that incomplete proteomes did not have a major influence on *Q*-values. The medium number of proteins with predicted signal peptides was 399 for the *Bacteroidetes*, 125 for *Chlorobi*, 163 for *Spirochaetes*, 128 for *Chlamydiae*, and 195 for *Cyanobacteria*. Removal of lipoproteins from the set of proteins with predicted signal peptides was a relatively “small correction.” The median values of the number of proteins with predicted type I signal peptides per species were 299 for the *Bacteroidetes*, 104 for *Chlorobi*, 146 for *Spirochaetes*, 112 for *Chlamydiae*, and 161 for *Cyanobacteria*. As lipoproteins have a Cys after the SPII cleavage site, their removal always increased predicted species *Q*-values.

### Cloning, Expression and Purification of Recombinant PgQC

The QC protein was expressed as a GST-tag fusion protein. Briefly, the entire coding region of *qc* (PG_2157) was amplified from a *P. gingivalis* W83 genomic template with Platinum Taq DNA Polymerase High Fidelity (Invitrogen) using primers F1_QC: ATTAGAATTCATGAAAAGACTGATAACAACAGGAG and R2_QC: ATTACTCGAGTCAGTGTGAAGCGGCTTTCACCTGTTCG. The 1001 bp PCR product was digested with *EcoRI – XhoI* and cloned downstream in-frame with the sequence encoding glutathione S-transferase (GST), into *EcoRI – XhoI* digested pGEX-6P-1 expression vector (GE Healthcare). Following confirmation by PCR, resulting expression pGEX/QC vector was transformed into *Escherichia coli* Bl21 (DE3) expression host. Transformed *E. coli* cells were grown in LB media at 37°C until OD_600_ 0.6, cooled down to 24°C and expression of recombinant protein was induced with 0.1 mM isopropyl-1-thio-β-galactopyranoside (IPTG). After overnight cultivation, cells were harvested by centrifugation (6,000 × *g*, 20 min), resuspended in PBS supplemented with lysozyme, and lysed by sonication (3 cycles of 10 × 3 s pulses at 17 W). Cell lysate was clarified by centrifugation (30,000 × *g*, 30 min) and loaded onto a pre-equilibrated glutathione-Sepharose^TM^ High Performance column. Recombinant GST-QC fusion protein was eluted using 50 mM Tris-HCl, pH 8.0, supplemented with 10 mM reduced glutathione. The purified GST-QC protein was subsequently incubated with PreScission^TM^ Protease (GE Healthcare) and subjected again to chromatography on glutathione-Sepharose^TM^ to remove the GST tag. The purity of the resulting protein was verified by SDS-PAGE electrophoresis (NuPAGE^R^ 4–12% Bis-Tris Gel, Invitrogen). Protein concentration was determined by BCA Assay (Sigma).

### PgQC Activity Assay

The activity of PgQC was determined essentially as described (33). Briefly, 150 μl of the assay buffer (40 mM Tris-HCl, 400 mM KCl, pH 8.0), 10 μl of chromogenic substrate (200 mM H-Gln-AMC in DMSO), and 10 μl of a recombinant bacterial pyroglutamyl aminopeptidase (25 U/ml, Unizyme Laboratories, Hørsholm, Denmark) were mixed together in a microtitration plate and preincubated for 10 min at 30°C. The reaction was initiated by addition of 30 μl of appropriately diluted purified rPgQC or *P. gingivalis* whole culture, or washed bacterial cells or subcellular fractions and after 1 min incubation the increase in fluorescence (λ_ex_ = 380 nm, λ_em_ = 460 nm) was recorded for 10–20 min at 30°C. Unspecific cleavage of the H-Gln-AMC substrate was determined by omitting pyroglutamyl aminopeptidase, the auxiliary enzyme. If necessary, the unspecific cleavage was subtracted from PgQC activity.

### *P. gingivalis* Culture and Cell Fractionation Procedures

*Porphyromonas gingivalis* culture fractionation was performed at 4°C as described previously (Nguyen et al., 2009) starting from stationary-phase (a 2-day-old) cultures adjusted to an OD_600_ of 1.5. Briefly, cells were collected by centrifugation at 6,000 × *g* for 15 min, washed once with phosphate-buffered saline (PBS), and resuspended in 5 ml of 0.25 M sucrose and 30 mM Tris, pH 7.6. After mixing gently for 10 min cells were repelleted at 12,500 × *g* for 15 min. The outer membrane was disrupted by the rapid addition of ice-cold distilled H_2_O and the spheroplasts were pelleted by centrifugation at 12,500 × *g* for 15 min. The supernatant was designated the periplasmic sample. The remaining spheroplast pellet was resuspended in 5 ml PBS and ultrasonicated in an ice-water bath. Cellular debris and membranes were pelleted by ultracentrifugation at 150,000 × *g* for 1 h, and the supernatant was designated the cytoplasmic sample. The remaining pellet was washed and resuspended in cold PBS by sonication. This fraction was designated the membrane sample.

For separation of individual membranes, washed cells were lysed by ultrasonication as described above. The membranes were pelleted by ultracentrifugation (150,000 × *g*, 1 h) washed with PBS to remove periplasmic and cytoplasmic proteins and resuspended in PBS by sonication. The inner membrane was dissolved with Sarkosyl (lauroyl sarcosine) and the residual Sarkosyl-resistant outer membranes (OM) were pelleted by ultracentrifugation (150,000 × *g*, 1 h). The supernatants were designated the IM samples while pellets washed and suspended by sonication in PBS were designated the OM samples. Purity of the various fractions was checked by Western blotting for A-LPS or gingipains and the biotin containing 15 kDa biotin carboxyl carrier protein (AccB alias MmdC or PG1609) as OM and IM specific markers, respectively (Nguyen et al., 2007; Lasica et al., 2016) (data not shown).

### Generation of *P. gingivalis* ΔRgpB Deletion Mutant

For generation of plasmids suitable for *rgpB* gene mutagenesis the pRgpBall master plasmid was first engineered based on the pURgpB-E construct (Nguyen et al., 2009). A partial *rgpB* gene section upstream of the erythromycin cassette was replaced with whole *rgpB* coding sequence, together with a 817 bp fragment containing its potential promotor. The new fragment was amplified with primers RgpBall_F and RgpBall_R using genomic DNA of *P. gingivalis* W83 and ligated into the linearized pURgpB-E plasmid (with EcoRI and SmaI restriction enzymes) by the Gibson’s method (Gibson et al., 2009) resulting in pRgpBall-erm plasmid. All primer sequences are listed in Supplementary Table 1.

Next, the RgpB deletional plasmid (pRgpBdel-erm) was obtained by the truncation of pRgpBall-erm plasmid using the PCR based SLIM method (Chiu et al., 2004) with primers listed in Supplementary Table 1. With this approach, only the small 3′ fragment (154 bp) of *rgpB* CDS was preserved in the pRgpBdel-erm plasmid. The ΔRgpB strain was obtained by a homologous recombination event: the plasmid was electroporated into the *P. gingivalis* W83 strain and the positive recombinant clones were selected with 5 μg/ml erythromycin. Proper recombination was verified by sequencing.

### Generation of *P. gingivalis* Gingipain Q Mutants

Mutagenesis of each gingipain required a dedicated master plasmid. For RgpB studies the pRgpBall-erm construct was used. The Q1N mutation (replacement cag codon into aac) was incorporated using the SLIM method (resulting in the pRgpBallQ1N-erm plasmid), the Q25A mutation (replacement of cag codon into gct) was introduced by the QuikChange method (Stratagene) (generating pRgpBallQ25A-erm), all sequences of applied primers are listed in Supplementary Table 1 in the RgpBQ1N section. These plasmids were introduced into the *P. gingivalis* W83 RgpA-C strain lacking the whole *rgpA* gene (Nguyen et al., 2009) by the electroporation and the recombined clones were selected with 5 μg/ml of erythromycin. The obtained strains were partially sequenced and named ΔRgpA/RgpBQ25N and ΔRgpA/RgpBQ25A, respectively.

For RgpA mutagenesis, a master plasmid pNRgpA-tet was engineered. Two DNA fragments were amplified from *P. gingivalis* genomic DNA. The upstream 915 bp fragment consisting of sequence directly adjacent to the RgpA promotor was amplified with primers RgpA_Up_F and RgpA_Up_R). A downstream 2835 bp fragment, comprising the 5′ sequence of the RgpA gene together with 388 bp of its proposed promotor was amplified with primers RgpA_Dw_F and RgpA_Dw_R. The tetracycline (*tetQ*) resistance cassette was amplified from the pT-COW plasmid (Belanger et al., 2007) with primers Tet_BamHI_F and Tet_SalI_R. The backbone, the pUC19 plasmid, was linearized by PCR reaction with primers puc_EcoRI_R and puc_HindIII_F. All four amplified fragments were combined in the single step reaction by the method described by Gibson et al. (2009). The Q24N mutation (replacement of the cag codon into aac) was incorporated to the construct with the SLIM method. Sequences of applied primers are listed in Supplementary Table 1 (RgpAQ24N section). This plasmid was introduced into the *P. gingivalis* W83 ΔRgpB strain lacking the whole *rgpB* gene by the electroporation and the recombined clones were selected with 1 μg/ml of tetracycline. The resulting strain was partially sequenced and named RgpBdel/RgpAQ24N. The non-mutated master plasmid was also introduced into the ΔRgpB strain *P. gingivalis* W83 and the unaffected expression and activity of RgpA was observed (data not shown).

For Kgp mutagenesis, the pNKgp-cep master plasmid was created in a similar manner. First, two fragments adjacent to the start of the hypothetical Kgp promotor were amplified from the genomic DNA, the 809 bp upstream fragment with Kg_Up_F and Kg_Up_R primers, while the 3271 bp downstream fragment with Kg_Dw_F and Kg_Dw_R primers. The beta-lactamase gene *cepA* was amplified with primers CepA_F and CepA_R from template synthetized by the Life Technologies based on the sequence deposited under AAA21538.1 number (Gene Bank). The pUC19 plasmid was linearized with primers pUC_SphI_R and pUC_BamHI_F. The Q20N mutation (replacement of the caa codon by aat) was incorporated to the construct by the SLIM method. Sequences of used primers are listed in Supplementary Table 1 (KgpQ20N section). This plasmid was introduced into the wild type *P. gingivalis* W83 by the electroporation and the recombined clones were selected with 2 μg/ml of ampicillin. Obtained strain was partially sequenced and named KgpQ20N. As a control, the non-mutated master plasmid was also electroporated into the *P. gingivalis* W83 strain and the unaffected expression and activity of Kgp was observed (data not shown).

### Gingipain Activity Assay

The amidolytic activities of Rgp and Kgp enzymes were assessed by the hydrolysis of the chromogenic substrate benzoyl-L-arginine-*p*-nitroanilide (BApNA) and carboxybenzoyl-L-lysine *p*-nitroanilide (zKpNA; Novabiochem, Germany), respectively. In a 96-well format, 20 μl samples were preincubated in assay buffer (200 mM Tris-HCl, 100 mM NaCl, 5 mM CaCl_2_ [pH 7.6] (Lasica et al., 2017), supplemented with fresh L-cysteine to 10 mM) for 2 min prior to the addition of 1 mM substrate in a total volume of 200 μl. For activity measurement of Sarkosyl-treated membrane fractionations, a 0.125 mM concentration of a synthetic arginine substrate pyro-glutamyl-glycyl-L-arginine-*p*-nitroanilide (pyroEGRpNA; Pharmacia-Harper, Uppsala, Sweden) was used instead of BApNA due to precipitation of the BApNA substrate in the presence of the Sarkosyl detergent. The presence of 0.1% Sarkosyl detergent in the assay did not affect the rate of substrate hydrolysis as determined with purified RgpB (data not shown). The rate of formation of *p*-nitroanilide was measured at 405 nm using a SpectraMax Plus spectrophotometer (Molecular Devices Inc., San Jose, CA, United States). For ease of comparison between mutants and statistical analyses of independent repetitions, activity units were defined as the total activity present in the RgpB^+^ control mutant culture equaling 100 U for culture partitioning studies, the total activity in the RgpB^+^ control mutant cells equaling 100 U for cellular fractionation studies, or the total activity in the RgpB^+^ control mutant membranes equaling 100 U for membrane fractionation studies.

## Results

### *P. gingivalis* SPI Substrates Typically Have a Q Downstream of the SPI Cleavage Site

The starting point for this study was the observation that many secreted *P. gingivalis* proteins with predicted signal peptides also had a glutamine (Q) residue immediately downstream. The generality of this observation was confirmed on a genome-wide basis using the batch version of SignalP (for gram-negative bacteria).

A Q immediately downstream of the signal peptide was predicted in about half of the cases. In the remaining cases, the residue after the signal peptide was frequently a cysteine, suggesting that these proteins were lipoproteins/SPII substrates. We used LipoP to identify and remove these proteins from the set. In the remaining set, which should only contain SPI substrates, the fraction of proteins with a Q after the SPI cleavage site exceeded 60%. Additional manual checks, including checks with earlier versions of the SignalP program, suggested that the true fraction of SPI substrates with a Q directly after the cleavage site may be even higher (Supplementary Data Sheet 1).

The enrichment of glutamine downstream of the SPI cleavage site did not appear to be specific for the proteins of a particular cellular compartment. CELLO (Yu et al., 2004) predictions identified SPI substrate proteins in the inner membrane, the periplasm, the outer membrane, and the extracellular space. In all compartments, the fraction of SPI substrate proteins with a Q immediately downstream of the SPI site was 48% or higher, clearly indicating that Q enrichment was not characteristic for proteins of a specific compartment (Supplementary Table 2). Placement of some SPI client proteins in the cytoplasm by the CELLO server suggests that some predictions are in error. Even with this reservation, Q enrichment does not appear to be characteristic for SPI client proteins in a particular compartment. This conclusion was further strengthened by the inspection of protein lists.

### Recombinant *P. gingivalis* PG2157 Has QC Activity and Resides in the Inner Membrane

The high frequency of newly exposed Q residues in SPI substrates in *P. gingivalis* and the previously reported detection of 7 *P. gingivalis* proteins with N-terminal pyroglutamate (Veith et al., 2002) suggested that glutamine cyclization might not be spontaneous, but enzymatically catalyzed. A BLASTP query of the *P. gingivalis* proteome with the human QC sequence suggested that PG2157 (also called PG_RS09565) may have QC activity. The recombinant protein (without signal peptide) did not exhibit aminopeptidase activity on any of the commercially available substrates of general formula NH_2_-L-Xaa-pNA or NH_2_-L-Xaa-AMC. However, it efficiently converted the fluorogenic substrate L-glutaminyl-AMC into its respective pyroglutamic acid derivative (*K*_m_ = 0.473 mM, *k*_cat_ = 0.356 s^-1^, *k*_cat_/*K*_m_ = 1.34 mM^-1^s^-1^). We therefore refer to PG2157 (PG_RS09565) as PgQC and to the recombinant version of the protein as rPgQC.

Sequence analysis of PgQC revealed a canonical signal peptide with the typical lipobox (Leu-Ser-Ala-Cys), suggesting that PgQC is a lipoprotein. Lipoproteins are translocated across the inner membrane via the Sec system, and initially anchored to the inner membrane by covalent attachment of a lipid anchor to the cysteine residue, followed by signal peptide cleavage, and typically *N*-acetylation. Some, but not all lipoproteins are subsequently transferred from the inner to the outer membrane. Therefore, we expected that PgQC should be anchored in the inner or outer membrane.

**FIGURE 1:**
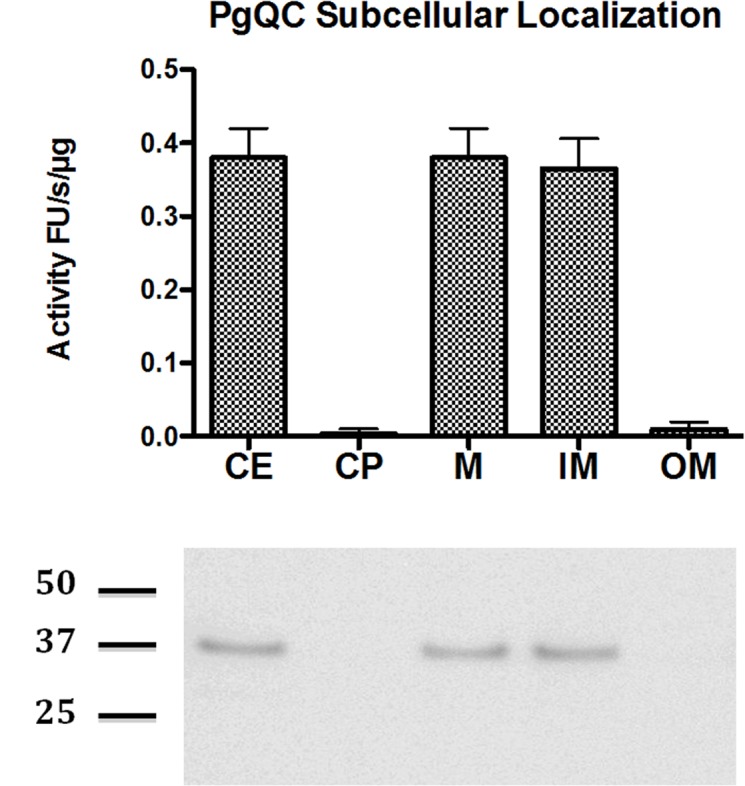
Distribution of QC activity and protein in *P. gingivalis. P. gingivalis* in the early stationary phase of growth was fractionated into sub-cellular fractions including whole lysed cell extract (CE), cytoplasm and periplasm (CP), total membranes (M), outer membrane (OM), and inner membrane (IM). QC activity was determined in each fraction standardized to the volume of washed cells suspended for sonication. The QC activity in each fraction was determined with L-Gln-AMC as a substrate using a coupled assay with pyroglutamyl aminopeptidase as an auxiliary enzyme. **(A)** The activity is shown as RF/s. Error bars indicate one standard deviation. **(B)** The presence of PgQC antigen in fractions was determined by western blot.

In order to determine QC localization experimentally, cell extract (CE) of *P. gingivalis* in late exponential/early stationary phase of growth was fractionated into cytoplasm and periplasm (CP), total membranes (M), outer membrane (OM), and inner membrane (IM). The purity of the membrane fractions was verified by the exclusive presence of the biotin-containing 15-kDa biotin carboxyl carrier protein (AccA alias MmdC or PG1609) and gingipains in the IM and OM, respectively (Veith et al., 2014) (data not shown). QC activity of the fractions was then measured using the enzyme-coupled assay already used previously to demonstrate the activity of the recombinant enzyme. QC activity was found in the IM and in fractions containing IM (CE and M) but not CP and OM (**Figure 1A**) clearly indicating that PgQC is anchored in the inner membrane. This localization was further confirmed by Western blot analysis of enriched subcellular fractions using rabbit polyclonal antibodies anti PgQC (**Figure 1B**).

### Pyroglutamate Is Present in Signal Peptidase I Substrates

In order to experimentally demonstrate pyroglutamate at the amino-terminus of SPI substrates, we reanalyzed mass spectrometry data for *P. gingivalis* outer membrane vesicles (Veith et al., 2013, 2014). Outer membrane vesicles (OMVs) are shedded continuously. Their vesicle lumen (VL), vesicle membrane (VM) and vesicle surface (VS) fractions contain proteins that are derived from the periplasm, the outer membrane, and extracellular proteins anchored to the outer membrane surface, respectively (Schwechheimer and Kuehn, 2015).

Pyroglutamate was inferred from the mass of the identified peptide, which is 17 Da less than what the unmodified peptide would be, and also by the fragmentation (MS/MS) pattern of the peptide. The MS/MS spectra of these peptides indicate that the -17 Da modification is present near the N-terminus of each peptide, most consistent with pyroglutamate formation. Altogether 27 proteins, all putative SPI substrates, with N-terminal pyroglutamate were identified (**Table 1**, last row, three proteins with pGlu and ambiguous location are omitted from the table). Interestingly, no semi-tryptic peptides with N-terminal glutamine were found in the entire *P. gingivalis* dataset, suggesting widespread pyroglutamate formation (**Table 1** and Supplementary Data Sheet 2).

**Table 1:** The table summarizes the statistics for potential and actual pyroglutamate (pGlu) formation in proteins that have been experimentally mapped to the vesicle lumen (VL), the vesicle membrane (VM), or the vesicle surface (VS) of outer membrane vesicles (OMVs).

	*P. gingivalis*			*T. forsythia*		
		
	VL	VM	VS	VL	VM	VS
Total	27	79	30	61	172	27
Q	19	36	24	n.d.	n.d.	n.d.
SIGNALP = Y	19	46	20	40	84	15
SIGNALP = Y & Q	10	19	16	27	31	12
pGlu detected	7	11	6	6	9	8


**Proteins from these compartments stem from the periplasm, the outer membrane, and the outer membrane surface, respectively. The row “Total” indicates the number of localized proteins, the row “Q” the number of proteins with a Q exposed by SPI signal peptide cleavage (according to a prior manual annotation, not focused on pyroglutamate formation), the row “SIGNALP = Y the number of proteins with signal peptide, and the row “SIGNALP = Y & Q” the number of such proteins exposing a glutamine after signal peptide cleavage. The row “pGlu detected” identifies the number of proteins with experimentally verified pyroglutamate. In very few instances, a pyroglutamate was detected in a protein not predicted to expose a glutamine after signal peptide cleavage due an erroneous SIGNALP prediction*.*

**FIGURE 2:**
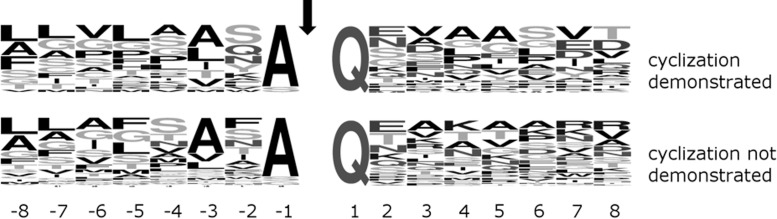
Comparison of (frequency mode) sequence logos around Q downstream of the signal peptide for *P. gingivalis* SPI client proteins of experimentally demonstrated **(top)** or undetermined **(bottom)** Q cyclization in OMVs. The signal peptidase motif A-X-A upstream of the SPI cleavage site (marked by an arrow) is clearly visible. The **(top and bottom)** logos are based on 25 and 38 sequences, respectively.

In order to further confirm this conclusion, we compared sequences around the cyclization site (and thus the SPI cleavage site) for proteins that were experimentally identified, had a glutamine after the predicted SPI cleavage site, and for which evidence for pyroglutamate formation was either available or not. The region upstream of the SPI cleavage site, normally not expected to influence QC, was included in the comparison in case SPI and QC may act together and QC preferences may be influenced by SPI preferences. However, we did not detect clear differences in the sequence logos of the two groups of proteins on either side of the critical Q residue, further supporting the conclusion that QC acts broadly and is not limited in its activity by specificity for residues adjacent to the substrate glutamine residue (**Figure 2**).

We also note that proteins with experimental evidence for pyroglutamate formation at the N-terminus were present in all compartments of the analyzed outer membrane vesicles, arguing against a role in partitioning proteins between VL, VM or VS of OMVs or the equivalent periplasmic space, outer membrane and outer membrane surface that these proteins stem from (**Table 1**).

### Q Downstream of the SPI Cleavage Site Affects RgpA, But Not RgpB and Kgp Secretion

In order to confirm that pyroglutamate formation does not affect secretion, we needed a model system in which pyroglutamate formation does not influence protein stability by controlling resilience against aminopeptidases. Gingipains RgpA, RgpB, and Kgp, the major proteolytic virulence factors of *P. gingivalis*, are good model systems in this respect. The proteins are initially expressed as preproproteins, and their type I signal peptides are then cleaved upon import into the periplasm, exposing N-terminal glutamine residues. The CTD-domain containing proteins are then exported further by the type IX secretion system (T9SS). Upon secretion from the periplasm to the outer membrane surface, the pro-regions are rapidly degraded, leaving only the mature forms that no longer contain the expected pyroglutamate N-terminal residue. This is an asset for comparing protein activity independent of issues of protein stability, but it unfortunately also prevents detection of the pyroglutamylation of the proregion in wild-type *P. gingivalis* strains. Nevertheless, we expect pyroglutamate formation based on its widespread occurrence (see above), and also because proRgpB is blocked for Edman degradation in a type IX secretion (T9SS) mutant that retains non-degraded proRgpB in the periplasm.

We used homologous recombination to construct *P. gingivalis* W83 strains expressing RgpAQ24N, RgpBQ25N, RgpBQ25A and KgpQ20N, in ΔRgpB, ΔRgpA and wild-type backgrounds, respectively. Due to overlapping RgpA and RgpB specificities (both enzymes cleave after arginine residues), RgpA activities had to be compared in ΔRgpB background, and *vice versa*, whereas Kgp activities could be compared in a wild-type background (for preparation of the strains, see Supplementary Figure 1). Mutation of the RgpA, RgpB, or Kgp glutamine after the SPI cleavage site did not alter *P. gingivalis* growth or the extracellular activity of prolyl tripeptidyl peptidase secreted by *P. gingivalis* independent of T9SS (data not shown). Gingipain activity was assayed in full cultures and the cell-free culture medium from cultures grown to the mid-exponential (OD_600 nm_ = 0.6–0.8), late exponential/early stationary (OD_600 nm_ = 1.2–1.4) and stationary (OD_600 nm_ > 2.0) phase of growth. We assayed either total extracellular activity, or separately the activities associated with the outer membrane surface and with the culture medium. In all cases, activities increased strongly over time from mid-exponential to late stationary cultures. Surprisingly, mutation of the Q after the SPI cleavage site had variable effects on the different gingipains.

**FIGURE 3:**
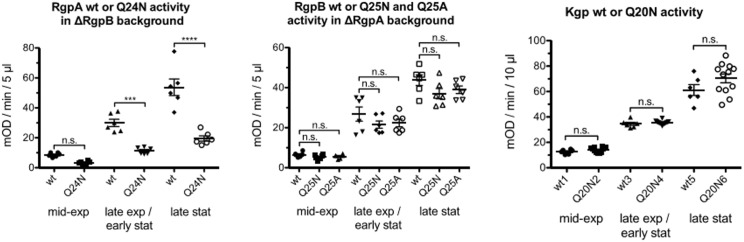
*Porphyromonas gingivalis* strains, ΔRgpB and ΔRgpB/RgpAQ24N **(left)**, ΔRgpA, ΔRgpA/RgpBQ25N and ΔRgpA/RgpBQ25A **(middle)** and wild-type and KgpQ20N **(right)** were grown in at least six independent cultures to mid-exponential (OD_600_ in the range 0.6–0.8), late exponential/early stationary (OD_600_ in the range 1.4–1.6) and late stationary phase (OD_600_ > 2) of growth then adjusted to the same OD_600_ of 0.6, 1.4, and 2, respectively. Gingipain activity was measured in whole cultures with appropriate substrates. One-way ANOVA tests were carried out separately for the three gingipains (degrees of freedom RgpA: 5,30; RgpB: 8,45; Kgp: 5,48). Significance was judged applying the Bonferroni correction for multiple hypotheses (conservatively assuming all against all comparisons, even though only comparisons between wild-types and mutants are of interest) for a *p* = 0.05 threshold. The ^∗∗∗^ symbol indicates *p* < 0.001, the ^∗∗∗∗^ symbol *p* < 0.0001.

The Q24N mutation in RgpA decreased overall activity several-fold, whereas the equivalent substitution Q25N (and also Q25A) in RgpB and Q20N in Kgp did not have a significant effect, irrespective of whether cells were assayed in mid-exponential, late exponential/early stationary or late stationary phase (**Figure 3**).

For all three gingipains, most of the activity was cell-associated, with only a minor contribution from protein in the medium. For RgpA, the Q24N decreased cell associated activity several fold, in all phases of growth. Activity in the medium was also significantly reduced in late stationary phase, the only growth stage with more than a marginal contribution from protein in the medium to the overall activity. In contrast, for RgpB, both the Q25N (and also Q25A) mutations altered the activity associated with cells and in the medium at most insignificantly. There was also no redistribution of activity between cells and medium. For Kgp, the Q20N substitution had an unexpected effect. Although the overall activity was not significantly altered, Q substitution increased activity in the medium at the expense of activity associated with cells, especially in mid-exponential phase, and to a lesser extent also in late exponential/early stationary phase (Supplementary Figure 2).

We suspect that the diverse effects of Q substitution must be related to complicated posttranslational processing of Kgp and RgpA leading to an assembly of large multidomain complexes of the catalytic and hemagglutinin-adhesion domains derived from initial polyproteins anchored into the OM via A-LPS (Pathirana et al., 2006) rather than *per se* translocation of the OM.

### High Q-Values Are Typical for *Bacteroidetes* species, But Not Related Phyla

In the following, we call the fraction of signal peptidase I substrates that have a glutamine immediately downstream of the SPI cleavage site the *Q*-value. The high *Q*-value is not unique for *P. gingivalis*, but is shared with other bacterial species. LipoP corrected SignalP predictions for various *Porphyromonas* species suggest *Q*-values between 59% (for *P. somerae*) and 77% (for *P. macacae*) (Supplementary Figure 3). Manual inspection of automatic prediction suggested that even the high Q enrichment values are still underestimates due to the mis-prediction of some signal peptide cleavage sites.

*Porphyromonas* species belong to *Bacteroidetes*, which in turn are placed in the FCB superphylum containing the *Fibrobacteres*, *Chlorobi*, and *Bacteroidetes*. In the *Bacteroidetes* group, 332 of 334 species (i.e., >99%) had predicted *Q*-values above 48%. Averaged across species, the *Q*-value was 71%, with a standard deviation for the variation between species of around 10% percent (outliers included). The two low *Q*-value outlier species (*Bacteroides pectinophilus*, 4% and *Candidatus cloacimonas* 24%) had a suspiciously low number of predicted proteins with signal peptides (21 and 83, compared to typically 300 in this group), suggesting a possible problem with sequencing or annotation rather than a genuine difference. CELLO predictions for *T. forsythia* confirm the conclusion for *P. gingivalis* that the enrichment of glutamine residues directly after the SPI cleavage site is not specific for proteins of a particular cellular compartment (Supplementary Table 2).

**FIGURE 4:**
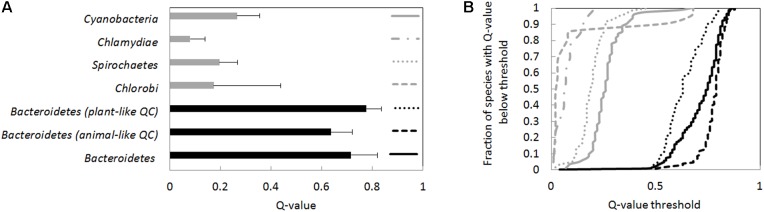
*Q*-values averages and *Q*-value distributions for different groups of bacteria. The *Q*-value was determined separately for every species in the groups. **(A)** Average *Q*-values for the species groups (species are given equal weight in the average), and the standard deviation of *Q*-values. *Bacteroidetes* have significantly higher *Q*-values than *Chlorobi*, *Spirochoaetes*, *Chlamydiae*, or cyanobacteria (*p* < 1E-6 for comparisons of *Bacteroidetes* against any of the other species groups according to the Wilcoxon rank sum test). **(B)** Cumulative *Q*-value distributions. For identification of species groups, refer to the symbols in the right panel (near *Q*-value 1). The *Q*-values for *Bacteroidetes* species are higher than for the other species groups also judging from cumulative distributions (*p* < 1E-6 according to the Kolmogorov–Smirnov test). Species were classified as having an animal- or plant-type QC according to BLASTP searches using the human and *A. thaliana* QC.

Across the entire FCB superphylum, Q-values were not consistently high. For most sequenced *Chlorobi* species, the predicted Q-value was below 9%. Three outliers were found in this group in little characterized bacteria (annotated *as Chloroherpeton thalassium*, *Q*-value 55%, *Chlorobi* bacterium, *Q*-value 63% and *Chlorobium* sp., *Q*-value 67%). Outside the *Bacteroidetes* and *Chlorobi*, a continuum of *Q*-values was found, ranging from 2% (*Chlorobaculum parvum*) to 77% (*Ignavibacterium album*), with no obvious pattern (**Figure 4**).

We also determined the cumulative *Q*-value distribution for selected bacterial phyla (**Figure 4**), thought to be relatively closely related to *Bacteroidetes* (Woese, 1987). Predicted *Q*-values for *Chlamydiae* and *Spirochaetes* species were typically below 20% and thus much lower than in *Bacteroidetes*. Cyanobacterial species typically also had lower *Q*-values. However, in a small fraction of cyanobacterial species (∼5%), *Q*-values were not much lower than those of *Bacteroidetes*. High *Q*-values were for example found for *Scytonema millei* (40%), *Leptolyngbya valderiana* (45%), *Hassallia byssoidea* (61%), and *Aphanocapsa montana* (67%) (**Figure 4** and Supplementary Data Sheet 1).

### *Bacteroidetes* Have Orthologs of Animal and Plant QCs

We searched complete bacterial proteomes in UNIPROT for orthologs of animal- and plant-type QCs. In a first step, we used the prototypical human and *Arabidopsis thaliana* QC sequences as queries. In a set of 1878 proteomes, we found 602 animal-type QCs, among them 402 (67%) in *Bacteroidetes*. We also identified 991 plant-type QCs, among them 401 (40%) in *Bacteroidetes*. As *Bacteroidetes* account for less than 10% of the proteome data (and even less in the redundant set), it is clear that QCs, particularly of the animal-type, are enriched in the *Bacteroidetes*. Orthologues of animal- and plant-type QCs tended to segregate according to phylogeny. Animal-type QCs were typically found in *Bacteroidia*, plant-type QCs in *Flavobacteriia*.

As the number of candidate QCs was smaller than the number of proteomes, we attempted to enlarge the set of candidate QCs by carrying out BLASTP queries with representative sequences from *Bacteroidia* and *Flavobacteriia*, the two largest groups within the *Bacteroidetes*. Starting from these sequences, and correcting for species duplicates, we identified 574 *Bacteroidetes* species containing animal-type and 507 *Bacteroidetes* species containing plant-type QC enzymes, but only 63 species containing both types of enzymes (*E*-value threshold 1E-4), compared to 1540 species in the duplication corrected proteome dataset.

We also attempted iterated searches, using representatives of CD-HIT identified sequence clusters (Makarova et al., 2006) to initiate additional searches. With this procedure, still more putative QCs were identified, but the concentration of hits in the *Bacteroidetes* phylum was reduced, likely because the set of putative QCs became contaminated by peptidases. Despite this complication, we can conclude from the simpler BLASTP searches that most and perhaps even all *Bacteroidetes* species have enzymes that could be suitable for glutamine cyclization. In the following, we focus on the candidate QC enzymes that can be demonstrated in a single BLASTP step with tight *E*-value threshold to be orthologous to the prototypical animal- or plant QC enzymes.

### The Orthologs of Animal and Plant-Like QCs in *Bacteroidetes* Have Intact Active Sites

In order to assess the chances that the animal- and plant-type QCs in bacteria were active, we checked alignments for the presence of key active site residues.

The prototypical animal QC is the human enzyme, HsQC. Its active site is built from E201 (involved in proton shuttling), D159, E202 (involved in binding the active site Zn^2+^ ion), and D248 (involved in both). In the *Bacteroidetes* orthologs of human QC, D159, E201 and D248 are highly conserved (>98%). In contrast, E202 is conserved only in 9% of *Bacteroidetes* orthologs, and replaced by an aspartate residue almost all remaining cases (exceptions < 1%). As aspartate and glutamate can both serve as Zn^2+^ ligands, we suspect that this substitution may not compromise activity, or may even be required to accommodate slight changes in the overall protein structure compared to HsQC. This conclusion is supported by the observation that an aspartate is also present in the 202 position in the PgQC enzyme, which we have already shown to be an active QC enzyme.

The prototypical plant QC is the enzyme from *C. papaya*, CpQC. Its active site is not as well understood as the active site of HsQC, but it is thought that E69, N155 (probably involved in proton shuttling) and N155, K225 and Q24 (probably involved in stabilizing the oxyanion intermediate) play a role in catalysis. Among the *Bacteroidetes* orthologues, E69, N155 and K225 are strictly conserved. In contrast, Q24 was conserved only in 81% of cases, and replaced by a glutamate in the remaining cases. The same substitution occurs in many plant enzymes, and also in the experimentally studied bacterial *Xanthomonas campestris* QC (XcQC). In this special case, the (natural) glutamate variant is still active, albeit an order of magnitude less so than the engineered glutamine variant (Huang et al., 2010), suggesting that both glutamine and glutamate in the active site are compatible with activity, although not necessarily at the same level.

We conclude from this analysis that *Bacteroidete*s orthologues of animal and plant QCs are likely to be active enzymes. This is directly suggestive of QC activity for plant QC orthologues, and compatible with either QC or aminopeptidase activity for animal QC orthologues. Classification of the enzymes as lipoproteins (like PgQC) could strengthen the case for QC activity.

### Most *Bacteroidetes* Orthologs of Animal and Plant QCs Are Predicted Lipoproteins

Among 401 orthologs of human QC in *Bacteroidetes*, 323 (∼80%) were predicted lipoproteins, 59 (∼15%) were predicted SPI substrates (∼15%), and the remaining 19 (∼5%) were predicted to be cytoplasmic. As orthologues of human QC are highly enriched in *Bacteroidetes*, relatively few were found in species not belonging to the *Bacteroidetes*. Among these, the fraction of predicted lipoproteins was much smaller. Only 38 (∼34%) enzymes were predicted to be lipoproteins, 40 (∼36%) were classified as SPI substrates, and remaining 34 (∼30%) as cytosolic proteins.

Among 430 orthologs of *A. thaliana* QC in *Bacteroidetes*, 376 were (∼87%) computationally classified as lipoproteins, 20 (∼5%) as SPI substrates, and 34 (∼8%) as cytoplasmic proteins. The predominance of predicted lipoproteins among plant-type QCs was much less pronounced when bacterial homologues of plant QC in general were considered. Among the 1000 bacterial proteins most similar to *A. thaliana* QC, predictions classified 475 (∼48%) as lipoproteins, 338 (34%) as SPI substrates, and 175 (18%) as cytosolic proteins.

Sensitivity (true positive rate, recall) of the LipoP algorithm for gram-negative bacteria has been reported to be around 90% (Juncker et al., 2003). The fraction of bacterial QCs predicted to be lipoproteins in non-*Bacteroidetes* species is much smaller, suggesting that not all are lipoproteins. In *Bacteroidetes*, the fraction of QC proteins predicted as lipoproteins comes close to the predicted sensitivity of the prediction algorithm. Thus, it appears likely that most if not all QCs in *Bacteroidetes* are lipoproteins, like the prototypical PgQC from *P. gingivalis*.

### Proteomic Datasets Confirm Glutamine Cyclization in Several *Bacteroidetes* Species

In order to confirm widespread pyroglutamyl formation in *Bacteroidetes*, and not only *P. gingivalis*, we analyzed additional data from previously reported proteomic studies (Veith et al., 2002, 2009, 2013, 2014, 2015). In addition to the already discussed 27 proteins from *P. gingivalis*, the collated data identify 27 proteins in *Tannerella forsythia*, 13 in *Parabacteroides distasonis*, 8 in *Prevotella intermedia* and 7 in *Cytophaga hutchinsonii* (Supplementary Figure 4 and Supplementary Data Sheet 2) with N-terminal pyroglutamate residue. N-terminal residues other than pyroglutamate were rare, as predicted from the bioinformatic studies.

Pyroglutamate was not detected at the amino-terminus of all SPI substrates that are predicted to expose an N-terminal glutamine residue after SPI cleavage (**Table 1**), most likely due to incomplete coverage and not due to selective pyroglutamate formation. As already reported for the *P. gingivalis* proteins, semi-tryptic peptides were never found to start with glutamine, even though internal tryptic peptides could be identified with N-terminal glutamine in both modified and unmodified states. Other circumstantial evidence also supports widespread rather than selective glutamine cyclization. We focused in particular on the *T. forsythia* data, which contained pyroglutamate evidence for the largest number of proteins in one species.

Experimentally detected proteins (by any peptide, not necessarily a semitryptic peptide) with glutamine after the SPI site were partitioned into proteins with and without evidence for glutamine cyclization. As already reported for *P. gingivalis*, amino acids around the Q were similar in the two groups, supporting broad QC specificity (Supplementary Figure 5).

Pyroglutamate detection is not consistent for paralog families in a single species. The *T. forsythia* proteome is rich in such families. Using CD-HIT to cluster proteins with a 30% identity threshold, we found at least three examples of paralog pairs with one or two members shown to have a pyroglutamate, and another member having a Q downstream of the signal peptide, without demonstration of glutamine cyclization (pyroglutamate in Tanf_04820 and Tanf_08875 versus no data for Tanf_05310, pyroglutamate in Tanf_11855 versus no data for Tanf_00065, pyroglutamate in Tanf_12915 versus no data for Tanf_07640).

Pyroglutamate detection for a protein of a given bacterial species is also not predictive for the ortholog in another species. In a first step, we used BLASTP (*E*-value threshold 10^-9^) to identify orthologous proteins in *T. forsythia* and *P. gingivalis* with experimentally verified localization. Only unique pairs were used, and proteins with more than one paralog in either species were excluded. Among the 41 pairs, 9 and 3 were found to have a pyroglutamate at the N-terminal end. Based on a random association alone, one would then expect experimental pyroglutamate demonstration for both proteins of a pair in (9^∗^3)/41 ∼ 0.7 cases. In fact, one such case was observed.

Together, the above observations are consistent with general glutamine cyclization, partially masked by incomplete mass spectrometry coverage (for example, due to low expression of some proteins, or because semi-tryptic peptides are too short or too long for efficient mass spectrometry detection). As already seen for *P. gingivalis*, pyroglutamate formation was not characteristic for proteins of a particular compartment, as *T. forsythia* proteins with pyroglutamate were also found in the vesicle lumen, in vesicle membranes, and on the vesicle surface of OMVs, which represent periplasmic, integral OM and cell-surface associated proteins, respectively (**Table 1**).

## Discussion

### Possible Prediction Bias Cannot Explain the Q Enrichment After SPI Sites

SignalP does not “look” specifically for a Q residue downstream of the signal peptide, but has been trained on proteins with experimentally determined localization. Therefore, we cannot formally exclude that prediction bias affects Q-values. However, we expect that if such bias exists at all, it cannot be severe. As *Bacteroidetes s*equences constitute only a small part of all sequences for gram-negative bacteria, and presumably also of the training set, their presence is unlikely to have significantly affected scoring rules. More importantly, for species not belonging to the *Bacteroidetes*, much lower *Q*-values are predicted using the exact same prediction rules, clearly indicating that high *Q*-values for *Bacteroidetes* are genuine. In fact, both manual inspection and tests with earlier versions of SignalP (Bendtsen et al., 2004) suggest that the reported *Q*-values are still underestimates.

### Despite Universally High *Q*-Values, There Are Two Separate QC Lineages in *Bacteroidetes*

High species *Q*-values are characteristic for *Bacteroidetes* and nearly universal in this phylum. Two separate evolutionary lineages of QCs are therefore surprising. *Q*-values tend to be higher for species that have a plant-like QC than for those that have an animal-like QC (average 78% and median 79% versus average 64% and median 63%) (**Figure 4**). Such a difference would be highly significant for independent species (Kolmogorov–Smirnov test, *p* < 3^∗^10^-8^). However, clades tend to share the QC type. Therefore, *Q*-value differences may reflect bacterial phylogeny, and might not be biologically meaningful.

### Localization of *P. gingivalis* QC to the Periplasmic Surface of the Inner Membrane Violates Lipoprotein Sorting Rules

All lipoproteins are initially anchored in the inner membrane, but some are later transported to the outer membrane by the Lol system (Zuckert, 2014). At least for *Escherichia coli*, a simple sorting rule, known as the “+2 rule” is thought to determine partitioning between the membranes. If the “+2 residue” is an aspartate (Yamaguchi et al., 1988), or one of several residues rarely found in this position (Seydel et al., 1999), the lipid anchor of these proteins remains in the inner membrane, otherwise it is flipped over to the outer membrane (Yamaguchi et al., 1988). The “+2 residue” of PgQC is an asparagine, which according to the “+2 rule” predicts anchoring in the outer membrane, in contrast to the experimental observation that demonstrates anchoring in the inner membrane.

The apparent contradiction is due to a breakdown of the “+2 rule” for *Bacteroidetes*. Using the OMV mass spectrometry data for *P. gingivalis* (Veith et al., 2004, 2014) and *T. forsythia* (Veith et al., 2009), we could demonstrate that aspartate was present at statistically expected frequencies or above in vesicle membrane anchored lipoproteins that correspond to outer membrane proteins, signaling a clear breakdown of the “+2 rule” (manuscript in preparation). Breakdown of the “+2 rule” in *Bacteroidetes* prevents predictions on whether the lipoprotein QCs from other *Bacteroidetes* species also localize to the inner rather than the outer membrane. At present, we consider anchoring in the inner membrane likely, in part because of the experimental findings for *P. gingivalis*, and in part because this localization “makes sense” for the apparently efficient cooperation with the inner membrane anchored SPI.

### Pyroglutamate Formation Is Widespread If Not Universal in Proteins Derived From the Periplasm, the Outer Membrane, and the Outer Membrane Surface

Detection of pyroglutamate in proteins from the vesicle lumen, vesicle membrane and vesicle surface of outer membrane vesicles shows that proteins that stem from the periplasm, the outer membrane and the outer membrane surface can have their N-terminal glutamine residues cyclized. The absence of semi-tryptic peptides with N-terminal glutamine, the very similar amino acid composition downstream of the N-terminal glutamine residue, and various other tests (inconsistent detection of pyroglutamate in paralogs of the same species, and between orthologs of different species), are all consistent with the idea that the modification is widespread, if not universal for proteins that are trafficked to the periplasm and expose an N-terminal glutamine residue after SPI cleavage. As more than half of SPI client proteins have a Q after the SPI cleavage site, this speaks for pyroglutamate at the amino terminus of the majority of these proteins in *Bacteroidetes*. It does not, however, necessarily suggest that the majority of secreted proteins in *Bacteroidetes* has an N-terminal pyroglutamate, since at least two secretion systems (T1SS and T6SS) bypass the periplasm and therefore also the QC enzyme.

### Pyroglutamate Formation Remains to Be Tested for Inner Membrane Proteins

In this study, we did not generate new mass spectrometry data, but only reanalyzed previously gathered mass spectrometry data. As inner membranes have not been extensively analyzed, the experimental data do not contain evidence for or against pyroglutamate formation at the amino terminus of inner membrane proteins. CELLO predictions place some proteins with type I signal peptide in the inner membrane. In both *P. gingivalis* and *T. forsythia*, the proportions of these proteins with a Q immediately after the SPI cleavage site are comparable or even higher than for the other secreted protein fractions, suggesting that glutamine cyclization is a possibility. Whether it actually takes place remains to be tested.

### Pyroglutamate Formation Does Not Control Type IX Secretion, Despite Phylogenic Co-occurrence

T9SSs have been reported to be present only in *Bacteroidetes*, but not *Chlorobi* (Abby et al., 2016). The striking phylogenetic coincidence between species with T9SS and those with high *Q*-values suggested a possible mechanistic link between the two. However, the coincidence may not be as clear-cut as originally thought, since BLAST searches suggest the possible occurrence of T9SSs in *Chlorobi*, *Fibrobacteres* and *Ignavibacteriae*, casting doubt on the previous conclusions (Abby et al., 2016). Moreover, T9SSs are known to be controlled by so-called C-terminal targeting domains (CTDs), which reside at the carboxyterminal ends of proteins (Lasica et al., 2017), making the involvement of the N-terminal ends unlikely, unless the termini are in proximity. With the exception of the findings for RgpA, the experimental data clearly argue against a role of glutaminyl cyclization in targeting to T9SSs.

### A Model for Pyroglutamate Formation in Proteins Destined to the Periplasm, the Outer Membrane, the Outer Membrane Surface, or the Medium

**FIGURE 5:**
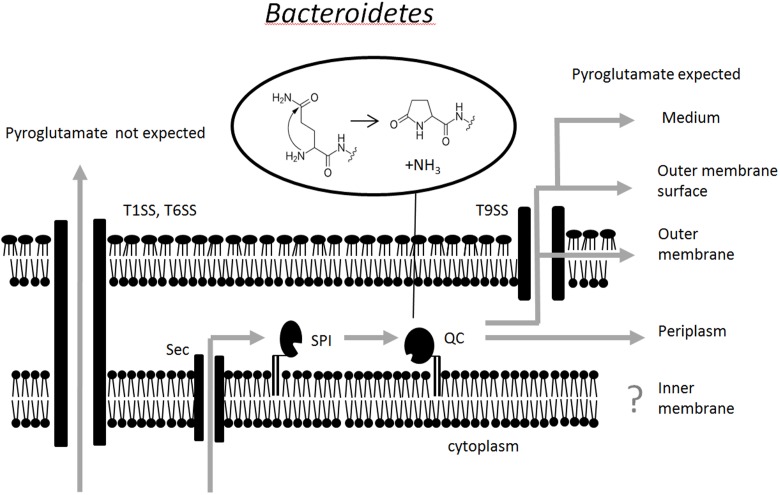
Schematic representation of the pathway for pyroglutamate formation at the amino terminus of proteins that transit to or through the periplasm. It is currently unclear whether or not SPI and QC are physically associated. Pyroglutamate formation has not yet been tested for proteins of the inner membrane.

Together, our data suggest a model for the processing of *Bacteroidetes* SPI substrates that reside in or transit through the periplasm. Their signal peptides are cleaved by SPI, a lipoprotein with active site on the periplasmic face of the inner membrane. Because of the enrichment of glutamine immediately downstream of the SPI cleavage site, this reaction typically exposes an amino-terminal glutamine residue. QC, another lipoprotein, also with active site on the periplasmic face of the inner membrane, is then ideally positioned to catalyze the cyclization of the glutamine residue to a pyroglutamate residue. The cooperation between SPI and QC is apparently efficient, suggesting either direct interaction or joint anchoring in lipid domains, which we have not yet tested. Pyroglutamate formation occurs for proteins that remain in the periplasm, as well as for proteins that are transported further into or through the outer membrane. We suggest the term “Q-rule” to describe the finding that glutamines are enriched after SPI cleavage sites, and that these glutamine residues are cyclized to form N-terminal pyroglutamate residues in proteins that are destined to the periplasm or beyond (**Figure 5**).

### Importance of the Q-Rule Pathway

The Q-rule pathway seems to be biologically important, not only judging by the number of proteins that are subject to the rule. *P. gingivalis* glutaminyl cyclase is present in both virulent and avirulent strains (Chen et al., 2004). According to an unbiased large scale transposon mutagenesis screen (Tenorio et al., 2011), the enzyme is essential even in laboratory culture conditions when *P. gingivalis* is not pitted against a host immune system. It is currently unclear why the enzyme is essential. Our data speak against a role of the modification in protein sorting. Given the host-associated lifestyle of many *Bacteroidetes* species, it is possible that glutaminyl cyclization protects secreted proteins against host aminopeptidases (excluding of course the host pyroglutamate aminopeptidases). However, this model does not explain why the Q-rule applies to SPI substrates that remain in the periplasm or why the glutaminyl cyclase is essential for *P. gingivalis* in culture conditions, unless *P. gingivalis* has become “addicted” to the Q-rule and now needs pyroglutamate formation for protection against its own periplasmic proteases as well.

## Author Contributions

DM and MN constructed and analyzed the mutant *P. gingivalis* strains. FV and JH analyzed the *P. gingivalis* QC activity and localization. PV and ER reanalyzed the mass spectrometry data. MB did the bioinformatics. MB and JP designed the project and wrote the manuscript with input from all the authors.

## Conflict of Interest Statement

The authors declare that the research was conducted in the absence of any commercial or financial relationships that could be construed as a potential conflict of interest.
