# Supplementary material for: The Bacteroidetes Q-Rule: Pyroglutamate in Signal Peptidase I Substrates
Source: Front Microbiol. 2018 Mar 1;9:230. doi: 10.3389/fmicb.2018.00230 (PMC5837995; doi:10.3389/fmicb.2018.00230)
Supplement: Supplementary file 1 [file Presentation_1.PDF]

Suppl. Tables and Figures for:

The *Bacteroidetes* Q-rule:

Pyroglutamate in signal peptidase I substrates

By Matthias Bochtler, Danuta Mizgalska, Florian Veillard, Magdalena Nowak,

John Houston, Paul Veith, Eric Reynolds, Jan Potempa

Suppl. Table 1:

**pRgpBall-erm plasmid**	
RgpBall_F	GACGGCCAGTGAATTCTTAACCATGCTGTGGTGACGAG
RgpBall_R	AGCGGAAGCTATCCCAACAGTCTCTTGGCGTAGTGCCAA
**pRgpBdel-erm plasmid**	
RgpBQ1N_Rs	AACGCCATTCCTCCCAACAG
RgpBdelFs	GGGATAGCTTCCGCTATTGCT
RgpBdelRt	GGGTCTGCCGGCTGTGCAAACGCCATTCCTCCCAACAG
RgpBdelFt	TGCACAGCCGGCAGACCCGGGATAGCTTCCGCTATTGCT
**RgpBQ24N mutation**	
RgpBQ1N_Fs	CGGTCGCAACCCACAAGTAC
RgpBQ1N_Ft	TGCAAACCCGGCAGAGCGCGGTCGCAACCCACAAGTAC
RgpBQ1N_Rs	AACGCCATTCCTCCCAACAG
RgpBQ1N_Rt	CGCTCTGCCGGGTTTGCAAACGCCATTCCTCCCAACAG
**RgpBQ25A mutation**	
Q25AF	GGAATGGCGTTTGCAGCTCCGGCAGAGCGCGGTC
Q25AR	GACCGCGCTCTGCCGGAGCTGCAAACGCCATTCC
**pNRgpA-tet plasmid**	
RgA_Up_F	GCCAGTGAATTCGGTCAGAGAGCCGA
RgA_Up_R	CGTTGTGGATCCTGAGCGTACCATATCTTTTAACC
RgpA_Dw_F	TTGGCAGTCGACTCGAGGAGCTGATTGGCTT
RgpA_Dw_R	TACGCCAAGCTTGAGGAGCAGCAATTG
Tet_BamHI_F	TCAGGATCCACAACGAATTATCTCCTTAAC
Tet_SalI_R	CGAGTCGACTGCCAAGTTCTAATGCTTC
puc_EcoRI_R	ACCGAATTCACTGGCCGTCGT
puc_HindIII_F	CTCAAGCTTGGCGTAATCATGGT
**RgpAQ25N mutation**	
RgpAQ1N F_s	ACGCAATCCGAATGTGAGATT
RgpAQ1N F_t	GAACCAGACAGAGTTGGGACGCAATCCGAATGTGAGATT
RgpAQ1N R_s	GCAAATGCCATTCCTCCTAAT
RgpAQ1N R_t	CCCAACTCTGTCTGGTTCGCAAATGCCATTCCTCCTAAT
**pNKgp-cep plasmid**	
Kg_Up_F	AGCTTGCATGCACACACCCCGAT
Kg_Up_R	ATGGAAGCTTAAGTCAGTCCAGCATGAGGAAG
Kg_Dw_F	ACTTGAGATCTTAACCTTGGTCTGCTCTAC
Kg_Dw_R	CCGGGGATCCTTCTACCGTAACGTC
CepA_F	GACTTAAGCTTCCATAGACGATGCCACACTG
CepA_R	GTTAAGATCTCAAGTCACCGATAGTGATAGTG
pUC_BamHI_F	TAGAAGGATCCCCGGGTACCGAGCT
pUC_SphI_R	TGTGTGCATGCAAGCTTGGCGTAATCAT
**KgpQ20N mutation**	
KgpQ1NFs	CTTGATGCTCCGACTACTCGA
KgpQ1NFt	AATAGCGCCAAGATTAAGCTTGATGCTCCGACTACTCGA
KgpQ1NRs	GGCGTAAAGACCAACTCCCA
KgpQ1NRt	CTTAATCTTGGCGCTATTGGCGTAAAGACCAACTCCCA

Suppl. Table 1: Primers used for construction of plasmids used for *P. gingivalis* mutagenesis.

Suppl. Table 2

	P. gingivalis			*T. forsythia*		
	**Q**	**Non-Q**	**Total**	**Q**	**Non-Q**	**Total**
Extracellular	10 (56%)	8	18	13 (39%)	20	33
OuterMembrane	29 (48%)	31	60	54 (58%)	39	93
Periplasm	18 (58%)	13	31	34 (43%)	46	80
InnerMembrane	8 (100%)	0	8	6 (60%)	4	10
Cytoplasm	25 (50%)	25	50	41 (49%)	43	84
All compartments	90 (54%)	77	167	148 (49%)	152	300

Suppl. Table 2: Proteins with or without P1' Q residue downstream of type I signal peptide according to their localization predicted by the CELLO program.

Suppl. Fig. 1

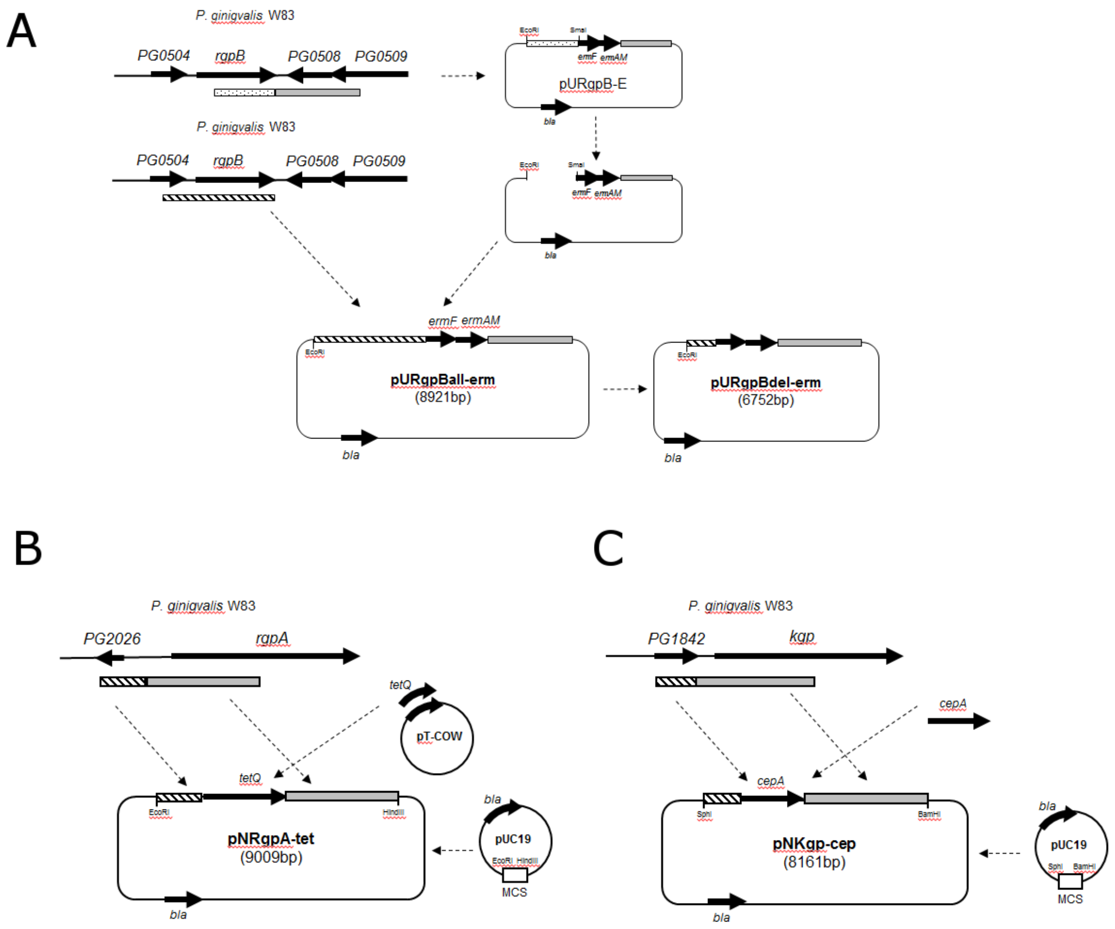

Suppl. Fig 1: Schematic representation of the steps undertaken to generate mutant *P. gingivalis* strains

Suppl. Fig. 2

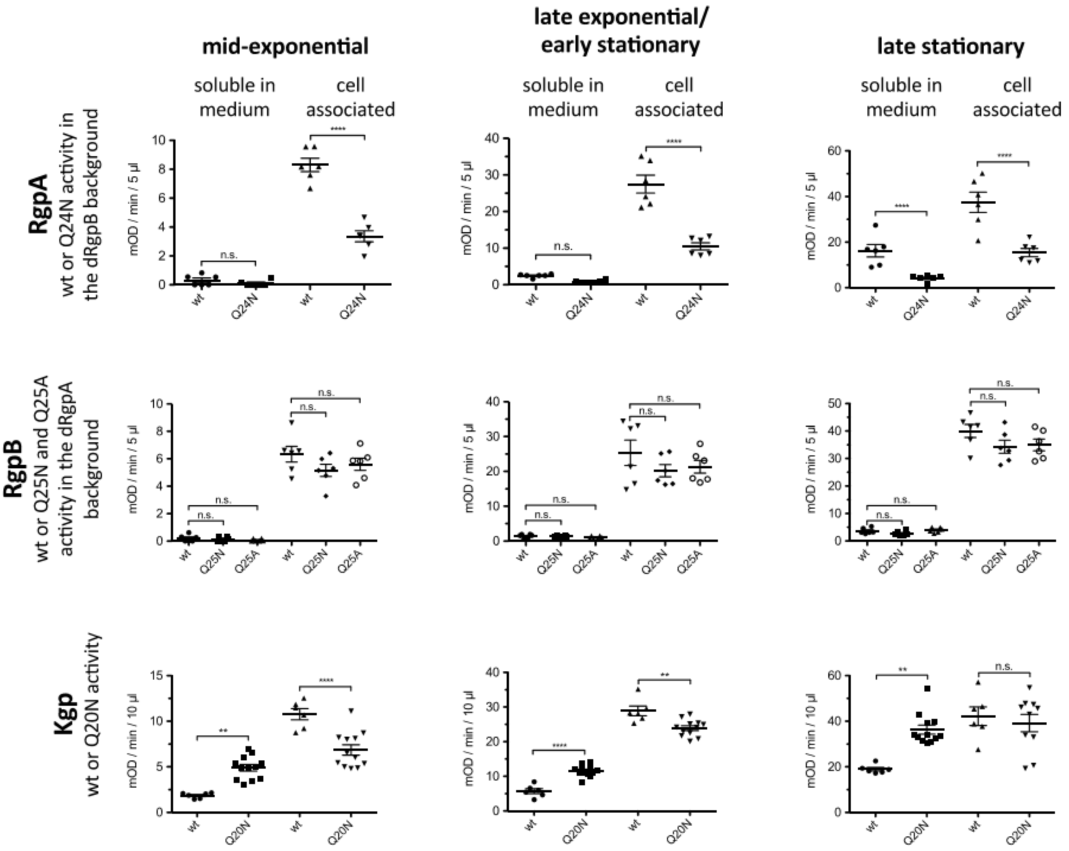

Suppl. Fig 2: *P. gingivalis* strains, ΔRgpA, ΔRgpA/RgpBQ25N and ΔRgpA/RgpBQ25A (top panels), ΔRgpB and ΔRgpB/RgpAQ24N (middle panels), and wild-type and KgpQ20N (bottom panels were grown in at least six independent cultures to mid-exponential (OD_{600} in the range 0.6-0.8), late exponential/early stationary (OD_{600} in the range 1.4-1.6) and late stationary phase (OD_{600} > 2) of growth then adjusted to the same OD_{600} of 0.6, 1.4 and 2, respectively. Gingipain activity was measured in whole cultures and culture media after cells were removed by centrifugation. Cell-associated activity was calculated by subtracting activity in medium from the total activity in whole culture. Graphs compare activity of gingipains released in the soluble form into growth media and cell-associated.

Suppl. Fig. 3

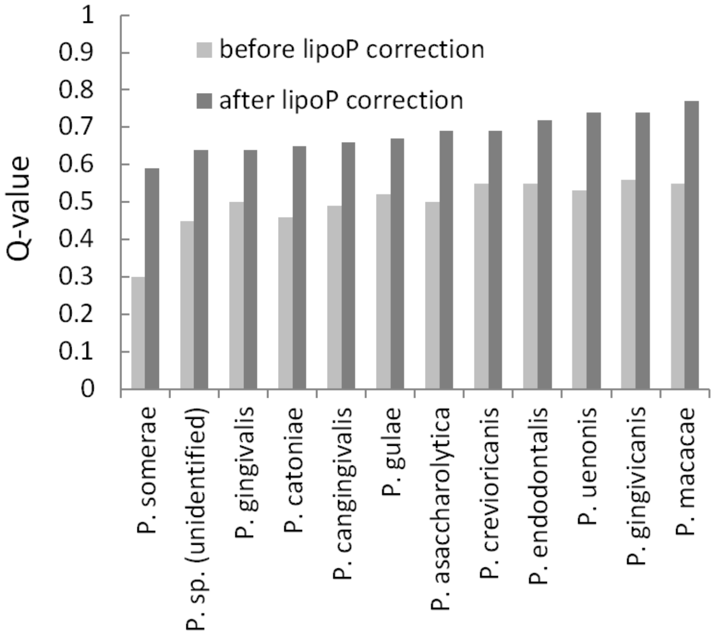

Suppl. Fig. 3: Fraction of predicted secreted proteins with a glutaminyl residue immediately downstream of the SPI cleavage site (Q-value) for different *Porphyromonas* species. Light grey bars are predictions based on SignalP alone, dark grey bars are calculated after removal of predicted lipoproteins, as identified by LipoP, from the set.

Suppl. Fig. 4

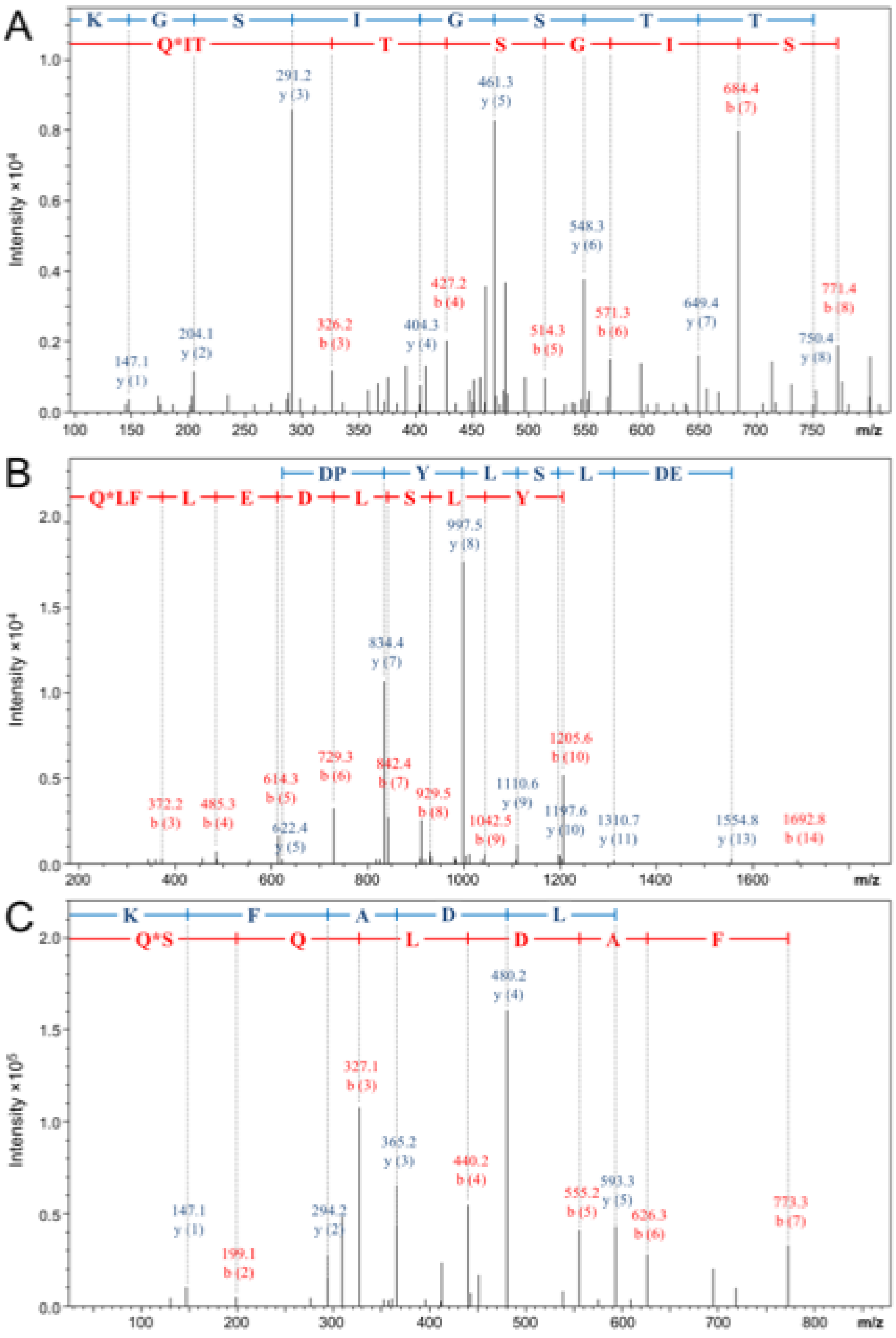

Suppl. Fig 4: Annotated MS/MS spectra of N-terminal peptides with pyroglutamate at C-terminus. A. MS/MS spectrum of Q*ITTSGISGK from an OmpA-related protein (PI1319) from *P. intermedia*. B. MS/MS spectrum of Q*LFLEDLSLYPDQFVTK from the *C. hutchinsonii* protein CHU_1281. C. MS/MS spectrum of Q*SQLDAFK from the *P. distasonis* protein BDI_1697. The N-terminal fragments (b-ions) are shown in red while the C-terminal fragments are shown in blue. Q* denotes pyroglutamate. Data for the parent ions can be found in Supplementary Table 1. These peptides were originally identified in a previous study (20).

Suppl. Fig. 5

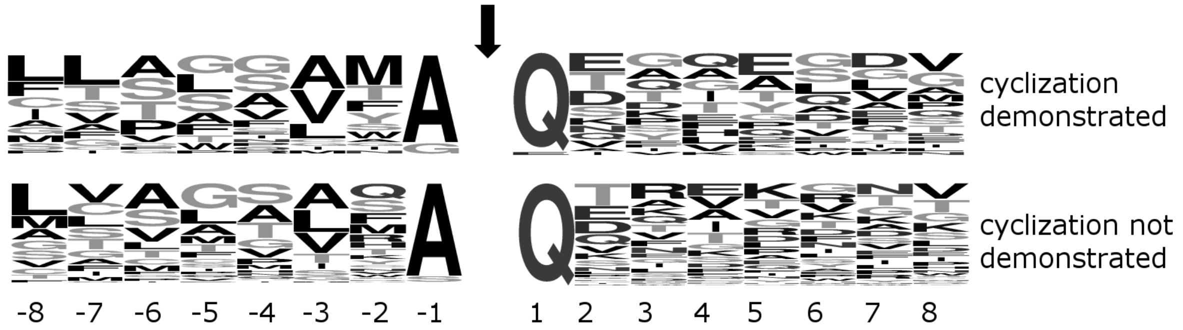

Suppl. Fig. 5: Comparison of (frequency mode) sequence logos around Q downstream of the signal peptide for *T. forsythia* SPI client proteins with experimentally demonstrated (top) or undetermined (bottom) Q cyclization in OMVs. The signal peptidase motif A-X-A upstream of the SPI cleavage site (marked by an arrow) is clearly visible. In one case, pyroglutamate in a T. forsythia protein results from glutamate rather than glutamine cyclization. This protein has been included in the calculation of the sequence logo. Only proteins with a Q after the predicted SPI site have been included in the comparison panel in the bottom. The top and bottom logos are based on 27 and 63 sequences, respectively.
